# Atomoxetine in abstinent cocaine users: Sex differences

**DOI:** 10.1016/j.dib.2017.08.011

**Published:** 2017-08-10

**Authors:** Elise E. DeVito, Aryeh I. Herman, Noah S. Konkus, Huiping Zhang, Mehmet Sofuoglu

**Affiliations:** a Department of Psychiatry, Yale School of Medicine, United States; b Veterans Affairs Medical Center, West Haven, CT, United States

## Abstract

Data presented are from a sex-differences secondary analysis of a human laboratory investigation of single doses of atomoxetine (40 mg and 80 mg) versus placebo in abstinent individuals with cocaine use disorders (CUD). Subjective drug effects, cognitive performance and cardiovascular measures were assessed. The primary atomoxetine dose analyses (which do not consider sex as a factor) are reported in full elsewhere (DeVito et al., 2017) [1].

**Specifications Table**

**Table:** 

Subject area	*Pharmacology*
More specific subject area	*Pharmacotherapy for Cocaine Use Disorder (CUD)*
Type of data	*Text file, table*
How data was acquired	*Human laboratory setting. Self-report questionnaires, computerized cognitive testing, heart rate and blood pressure measurements.*
Data format	*Analyzed*
Experimental factors	*Within-subject fixed factor of ‘dose’ (placebo, 40 mg, 80 mg atomoxetine); between-subject factor of ‘sex’ (male, female)*
Experimental features	*Double-blind, placebo-controlled, within-subject cross-over design with one pill condition (placebo, 40 mg or 80 mg atomoxetine) on each test day, with washout time between test days. Participants met criteria for cocaine use disorder but were abstinent from cocaine for 1-12 months.*
Data source location	*West Haven, CT*
Data accessibility	*Data is not available in a public repository.*

**Value of the data**

• There are no pharmacotherapies currently approved for the treatment of CUD. Pharmacotherapies with low abuse potential but cognitive enhancing properties could be effective adjunct therapies for CUD, since poorer cognitive function has been linked with worse clinical outcomes including risk of relapse [2], [3], [4], [5], [6], [7].
• A norephinephrine transporter inhibitor, atomoxetine, has been proposed as a candidate treatment for CUD, based on its efficacy as a cognitive enhancer in other clinical populations [8], [9] and its impact on addictive processes in preclinical [10], [11], [12], [13], [14] and human laboratory studies [15], [16], [17].
• Sex is a potentially important factor which could contribute to individual variability in response to medication [18]. Sex differences have been demonstrated in the etiology, disease course, health consequences and treatment response of CUDs (e.g. [19], [20], [21], [22], [23]), including poorer clinical outcomes for women in trials of medications for CUDs (e.g. disulfiram [24], modafinil [25], naltrexone [26]). There is mixed evidence of sex differences in response to atomoxetine for attention deficit hyperactivity disorder (ADHD) ([27], but see also [28]). Preclinical research indicates greater efficacy for atomoxetine in males than females [29], [30]. Although data specifically testing sex differences in atomoxetine response is limited, convergent evidence underlines the likelihood of sex-sensitive effects of noradrenergic medication for substance use disorders (e.g. [31]).
• The sample size in the current data was underpowered to investigate sex differences properly. These preliminary data may be hypothesis-generating for future studies of atomoxetine in men and women with CUD.

## 1 Data

Data presented are from a secondary analyses of sex differences. Data are subjective drug effects, cardiovascular response, and computerized cognitive task outcomes in abstinent individuals with cocaine use disorder (CUD) who received placebo, 40 mg atomoxetine and 80 mg atomoxetine. The detailed methods and primary dose effects analyses from this dataset are reported elsewhere [1].

Baseline and demographics data are reported in full in Table 1. Data for the effects of atomoxetine by sex on cardiovascular responses, subjective drug responses, mood and cognitive function are reported in Table 2. Briefly, treatment-by-sex interactions on drug-like effects showed more positive (euphoria) and less negative (dysphoria, sedation) subjective effects in women relative to men in response to the medication versus placebo. For mood, treatment-by-sex interactions reflected atomoxetine dose-related decreases in ‘fatigue’ and ‘depression’ in men, but increases in women. Furthermore, treatment-by-sex interaction indicated reduced ‘tension’ at the low-dose (relative to placebo or high-dose) condition, in men but not women. In terms of cognitive function, a treatment-by-sex interaction on Immediate Memory Task (IMT) revealed dose-related improvements (e.g., faster correct responses) in men, but a trend of dose-related decrements in performance in women.

**Table 1:** Baseline measures by sex.

**Measures**	**Sex**					
	**Male (*N*=29)**		**Female (*N*=10)**		**Statistics (by Sex)**	
	*N*	*(%)*	*N*	*(%)*	*Wald*	*(p)*
**Demographics**						
Race					1.37	0.242
African American/Black	14	48.28%	7	70.00%		
Not of Hispanic Origin	14	48.28%	7	70.00%		
Hispanic Origin	0	0.00%	0	0.00%		
European American	15	51.72%	3	30.00%		
Not of Hispanic Origin	14	48.28%	1	10.00%		
Hispanic Origin	1	3.45%	2	20.00%		
Highest level of completed education					**5.34**	**0.021**
College/University graduate	1	3.45%	0	0.00%		
Partial college training	10	34.48%	1	10.00%		
High School graduate/GED	17	58.62%	6	60.00%		
Partial high school	1	3.45%	3	30.00%		
Marital status					1.85	0.605
Never married	16	55.17%	6	60.00%		
Married	5	17.24%	1	10.00%		
Separated	2	6.90%	2	20.00%		
Divorced	6	20.69%	1	10.00%		
Employment status					1.42	0.492
Full-time (35 or more hours per week)	5	17.24%	2	20.00%		
Unemployed less than one month	2	6.90%	2	20.00%		
Unemployed greater than one month	22	75.86%	6	60.00%		
Sex					N/A	N/A
Male	29	100.00%	0	0.00%		
Female	0	0.00%	10	100.00%		
						
	*Mean*	*(SD)*	*Mean*	*(SD)*	*F*	*(p)*
Age, years	40.93	7.62	42.00	7.36	0.15	0.702
**Self-reported measures at baseline**						
CES-D summary score	8.95	7.15	8.20	5.63	0.09	0.766
CTQ						
Physical Abuse	7.79	4.19	10.40	5.76	2.35	0.134
Physical Neglect	7.79	3.16	9.60	4.62	1.91	0.176
Emotional Abuse	8.45	4.70	10.00	4.27	0.85	0.364
Emotional Neglect	9.93	5.44	11.22	4.94	0.40	0.530
Sexual Abuse	7.00	4.89	7.90	4.77	0.25	0.617

Statistics (F(p)) or Wald (p) as appropriate, are reported. Results reaching the statistical significance at *p*≤0.05 level are considered statistically significant (bold). CES-D: Center for Epidemiologic Studies Depression Scale; CTQ: Childhood Trauma Questionnaire; SD: Standard Deviation.

**Table 2:** Sex analyses.

	**Sex**												**Statistic**		
**Outcome Measures**	**Women (*N*=10)**						**Men (*N*=29)**						**Dose**	**Sex**	**Sex by Dose**
	**Placebo**		**40 mg**		**80 mg**		**Placebo**		**40 mg**		**80 mg**		*F(p)*	*F(p)*	*F(p)*
	*Mean*	*SD*	*Mean*	*SD*	*Mean*	*SD*	*Mean*	*SD*	*Mean*	*SD*	*Mean*	*SD*			
**Cardiovascular**															
Heart Rate	73.53	9.05	78.20	9.99	78.53	12.55	71.69	10.69	74.51	11.50	76.57	12.66	30.51 (<0.0001); 40,80ATX>PLA	–	–
Systolic BP	118.60	10.15	122.53	11.89	121.58	11.29	119.92	11.07	123.17	12.65	124.03	11.98	10.45 (<0.0001); 40,80ATX>PLA	–	–
Diastolic BP	71.80	8.30	72.97	7.80	74.07	7.89	71.03	8.86	72.45	11.23	73.17	10.19	5.35 (0.005); 80 ATX>PLA	–	–
**Subjective drug effects**															
*ARCI*															
Sedation (PCAG)⁎	4.83	3.64	4.49	3.16	4.05	3.02	3.13	2.02	2.97	1.88	3.62	2.86	–	–	4.77 (0.009); M: 80ATX>PLA,40ATX; W: NS
Dysphoria (LSD)⁎	3.08	1.94	3.33	1.75	3.08	1.62	2.44	1.27	2.64	1.57	3.25	2.15	–	–	3.45 (0.033); M: 80ATX>PLA,40ATX; W: NS
Euphoria (MBG)⁎	5.18	4.68	5.87	4.62	6.40	4.63	6.31	4.80	6.02	4.73	5.49	4.29	–	–	3.92 (0.021); M: NS; W: 80ATX>PLA
Stimulant-Like Effects (A)	3.93	2.48	4.10	2.72	4.28	2.72	4.10	2.58	4.23	2.48	4.03	2.34	–	–	–
Intellectual Efficiency and Energy (BG)	6.63	2.87	6.64	3.01	6.78	3.17	6.83	2.28	6.82	2.01	6.42	2.31	–	–	–
*DEQ*															
Feel Good Factor	0.67	0.93	0.87	1.13	0.62	0.86	0.68	1.06	1.12	1.30	0.88	1.01	5.92 (0.003); 40ATX>PLA, 80ATX	–	–
Negative Factor	0.53	0.89	0.69	0.81	0.61	0.80	0.62	0.71	0.86	0.89	0.78	0.85	5.40 (0.005); 40 ATX>PLA	–	–
Stimulatory Factor	0.60	0.83	1.19	1.10	1.03	1.17	0.89	1.16	1.22	1.65	1.12	1.09	9.75 (<0.0001); 40,80ATX>PLA	–	–
															
**Mood**															
*POMS*															
Anger	1.18	1.39	1.43	1.93	1.73	1.68	2.25	1.65	2.60	2.17	2.43	2.54	–	–	–
Depression	2.50	2.00	2.65	2.00	3.15	2.24	3.84	2.55	3.63	2.37	3.81	2.61	–	4.28 (0.045); M>W	–
Fatigue⁎	4.22	3.11	3.83	2.84	4.88	2.92	6.63	3.11	6.11	3.24	6.17	3.03	4.27 (0.015); PLA,80ATX>40ATX	6.67 (0.013); M>W	3.54 (0.030); M: PLA>40,80ATX; W: 80ATX>40ATX; M>W at PLA, 40ATX but not 80ATX
Confusion	4.73	0.88	5.04	1.47	4.70	1.02	4.48	1.25	4.67	1.20	4.64	1.55	–	–	–
Tension⁎	3.83	1.89	4.38	2.25	4.25	2.32	5.59	2.06	5.44	2.22	5.62	2.01	–	8.70 (0.005); M>W	3.24 (0.040); M: PLA>40ATX; W: NS; M>W at PLA, 80ATX but not 40ATX
Vigor	2.13	2.19	2.20	2.42	2.70	2.20	2.69	1.64	2.90	2.11	2.97	2.19	–	–	–
**Cognitive**															
*IMT*															
Discriminability (d')	0.70	0.52	0.86	0.95	0.73	0.70	1.16	0.82	1.24	0.96	1.29	0.91	–	–	–
Response Bias (Beta)	1.21	0.36	1.77	2.25	1.16	0.39	1.13	0.48	1.18	0.61	1.09	0.65	–	–	–
Mean Correct RT⁎	537.35	75.56	556.96	63.48	540.41	55.84	544.67	84.88	520.09	86.73	530.83	90.67	–	–	3.84 (0.026); M: PLA>40ATX; W: NS
*RVP*															
Discriminability (A')	0.87	0.05	0.87	0.04	0.88	0.07	0.87	0.11	0.89	0.06	0.89	0.07	–	–	–
Response Bias (B'')	0.77	0.19	0.81	0.18	0.82	0.20	0.85	0.23	0.89	0.12	0.87	0.14	–	–	–
Mean Correct RT	520.65	103.83	520.01	126.21	565.78	183.42	442.82	110.75	420.52	87.35	410.74	78.63	–	–	–
*SST*															
SSRT	255.78	155.57	250.90	174.04	292.30	156.95	215.87	75.00	211.07	79.29	192.99	84.54	–	–	–
Median Correct RT	688.90	227.89	653.95	164.26	642.05	203.94	656.69	169.64	657.45	212.92	636.59	186.16	–	–	–
Mean Correct RT	742.82	242.90	721.26	186.97	698.29	218.80	735.18	222.37	724.19	256.57	684.82	197.88	–	–	–
SD of Correct RT	318.79	171.65	391.27	300.21	320.29	248.84	437.87	417.17	384.95	301.41	290.72	198.74	–	–	–

Raw means and standard deviations are reported by sex. ATX: atomoxetine; PLA: Placebo; SD: Standard Deviation; POMS: Profile of Mood States; ARCI: Addiction Research Center Inventory; DEQ: Drug Effects Questionnaire; BP: blood pressure; RT: response time (ms); SSRT: Stop-Signal Reaction-Time; RVP: Rapid Visual Processing; SST: Stop Signal Task; IMT: Immediate Memory Task.

Missing data by sex and dose visit: No missing data for women (*N*=10 at each dose visit); Men Placebo visit (5 missing DEQ, ARCI, Physiological; 4 missing SST, RVP; 3 missing IMT); Men 40ATX visit (2 missing RVP, SST, IMT); Men 80ATX visit (3 missing DEQ, ARCI, POMS, Physiological, Cognitive).

Statistics that did not reach at least significance level of *p*<0.05 are not reported (indicated by '-')

⁎ Indicates dose by sex interaction effect (uncorrected *p*<0.05).

## 2 Experimental design, materials and methods

Methods are described in detail elsewhere [1]. Participants were otherwise healthy individuals (*N*=39) who met diagnostic criteria for cocaine dependence in early remission (i.e., abstinent >30 days, <1 year).

In this randomized, double-blind, placebo-controlled, within-subject crossover design participants received 40 mg, 80 mg atomoxetine, and placebo treatment, one pill per day, over three test days. Test days were scheduled approximately 6 days apart. Order of treatment condition (across test days) was randomly assigned and counter-balanced across individuals.

Outcome measures included cardiovascular (heart rate, blood pressure), subjective drug effects (Drug Effects Questionnaire (DEQ) [32]; Addiction Research Center Inventory (ARCI) [33]), mood Profile of Mood States (POMS) [34], and cognitive tasks measuring memory, attention and response inhibition performance (Immediate Memory Task (IMT) [35], [36], [37]; CANTAB Rapid Visual Information Processing (RVP) [38]; CANTAB Stop Signal Task (SST) [39]).

For demographic and baseline data, men and women were compared using analysis of variance (ANOVA) for continuous, or logistic regression for categorical variables. A mixed-effect repeated-measures analysis assessed in JMP (version 11.0) for treatment effect; including a fixed main effect for treatment (placebo, 40 or 80 mg atomoxetine), a random effect for participant, and a between subject factor of sex (men, women) and sex by treatment effect interaction term. When data was collected across multiple time points, all post-pill administration time-points were included in the analyses. To account for possible carryover effects of the medication or learning/test-retest effects across testing days, analyses were re-run including test day (1,2,3) and test day interactions. Findings (i.e., significance level) remained stable with and without test day, therefore data are presented from the simpler analysis excluding day. Bonferroni corrections were applied for the number of outcomes tested within each domain (cardiovascular, subjective drug effect, mood, cognition). Reported data survive Bonferroni corrections unless otherwise stated.
