# Supplementary material for: Atomoxetine in abstinent cocaine users: Sex differences
Source: Data Brief. 2017 Aug 10;14:566–72. doi: 10.1016/j.dib.2017.08.011 (PMC5568877; doi:10.1016/j.dib.2017.08.011)
Supplement: Supplementary file 1 — Transparency document [file mmc1.docx]

**Declaration of interest**

Authors report no related conflicting interests.
